# A Storage-Dependent Platinum Functionalization with a Commercial Pre-Polymer Useful for Hydrogen Peroxide and Ascorbic Acid Detection

**DOI:** 10.3390/s19112435

**Published:** 2019-05-28

**Authors:** Patrizia Monti, Quirico Migheli, Andrea R. Bartiromo, Antonio Pauciulo, Rocco Gliubizzi, Salvatore Marceddu, Pier A. Serra, Giovanna Delogu

**Affiliations:** 1 Dipartimento di Agraria and Unità di Ricerca Istituto Nazionale di Biostrutture e Biosistemi, Università degli Studi di Sassari, Viale Italia 39, I-07100 Sassari, Italy; pmonti@uniss.it (P.M.); qmigheli@uniss.it (Q.M.); 2 BI-QEM Specialties SpA, R&D Department – Zona Industriale, I-84021 Buccino (SA), Italy; andrea.bartiromo@bi-qem.com (A.R.B.); antonio.pauciulo@bi-qem.com (A.P.); 3 Istituto CNR di Scienze delle Produzioni Alimentari, Traversa La Crucca 3, I-07100 Sassari, Italy; salvatore.marceddu@ispa.cnr.it; 4 Dipartimento di Medicina Clinica e Sperimentale, Università degli Studi di Sassari, Viale S. Pietro 43/b, I-07100 Sassari, Italy; 5 Istituto CNR di Chimica Biomolecolare, Traversa La Crucca 3, I-07100 Sassari, Italy; giovanna.delogu@icb.cnr.it

**Keywords:** modified poly-dihydroxydiphenyl sulfone, self-rejection, sensor, commercial sulfones

## Abstract

A preliminary assessment of properties of the commercial product Chemiplus 2DS HB (BI-QEM Specialties SpA) is proposed. Cyclic voltammetry of this oligomer containing sulfate/sulfone groups shows a single oxidative peak at +0.866 V vs. Ag/AgCl, and its passivating process on Pt electrode suggests the formation of a non-conductive layer. Electrode modification was achieved by exploiting the constant potential amperometry setting potential at +0.900 V vs. Ag/AgCl. A substantial change in the oxidative currents from electroactive species H_2_O_2_ and ascorbic acid (AA) were observed on Pt/Chemiplus 2DS HB sensors compared to unmodified Pt. Furthermore, the influence of different storage conditions on modified sensors was examined. A storage solution containing AA concentration from 0.1 until 10 mM maintained effective AA rejection of Pt/Chemiplus 2DS HB after 7 days from construction; H_2_O_2_ oxidation capability was also retained. Sulfone and sulfonate groups of Chemiplus 2DS HB are likely responsible for the dimensionality of the film and the electrostatic interaction leading to a self-blocking/self-rejection of AA. The way Pt/Chemiplus can reveal the AA presence depends on the maintaining of AA rejection, and this peculiarity can distinguish it from other sensors or biosensors.

## 1. Introduction

Ascorbic acid (AA) is a small organic molecule of extraordinary interest; it has a plethora of metabolic functions in both plants and animals [1]. As Vitamin C, it represents an essential nutrient for human diet and health [2]. Because of its properties, it is frequently added to food and drinks under E300 additive [3] in order to improve nutritional value, or to preserve technical or aesthetic features of fresh or processed food. AA is also being used in pharmaceuticals [4], as a preservative in the formulation of cosmetics [5], and as additive in engineered tissues [6].

Quantitation and detection of AA is achieved by different analytical methods, mainly by mass and UV spectroscopy connected to liquid chromatography. Nevertheless, both analytical methods suffer from preliminary elaboration of the matrix. An inexpensive and effective way to assess AA over a wide range of matrices involves the use of sensors [7], biosensors, and electro-analysis [8,9,10].

When the detection technique is electrochemical, AA can be also regarded as an interferent. In fact, the analytical signal can be seriously affected by AA oxidation, a process that occurs at relatively low potential in sensors based on carbon or Pt. AA interference must be overcome, especially when a large overpotential is required, for example, by oxidation of H_2_O_2_. The use of polymeric films can be helpful for both biosensors and sensors. In amperometric oxidase-based biosensors, electro-deposited films prevent AA from reaching the electro-catalytic surface [10,11]. In electrochemical sensors based on metal nanoparticles, the use of anionic polymeric Nafion helps AA rejection by improving H_2_O_2_ detection [12]. These polymers are evaluated for their transparency regarding H_2_O_2_ and their efficiency at rejecting AA; in addition, the permeability of these probe molecules through the membrane should be stable over time and under all working conditions.

Among anionic polymers applied in the manufacturing of sensors, the use of membrane-containing sulfone/sulfonic groups has been reviewed [13]. These polymers are characterized by high strength, good electrical characteristics, transparency, resistance to greases, and the presence of many solvents and chemicals. Their application spans from sensor applications [14], to enzyme immobilization [15], to biosensors [16,17]. Nafion is one of the most common and commercially available polymer, and has ionic properties due to the presence of a sulfonate group that is able to reject small, negatively charged interfering species such as AA. The degrees in sulfonation of membranes may alter hydrophilicity and ion permeation, enhancing the performance of the sensor device [18]. Poly-sulfone films are impermeable to charged molecules such as uric acid and ascorbic acid, and are permeable to hydrogen peroxide [19]. Poly-sulfonated membranes are characterized by the presence of larger surface pore size and higher sub-layer porosity in comparison with polysulfone membranes [20,21]. In virtue of the unique features of sulfone/sulfonated membranes, research on polymers embedded with sulfone and sulfonate groups as permselective films in biosensor manufacturing has become increasingly important.

**Figure 1:**
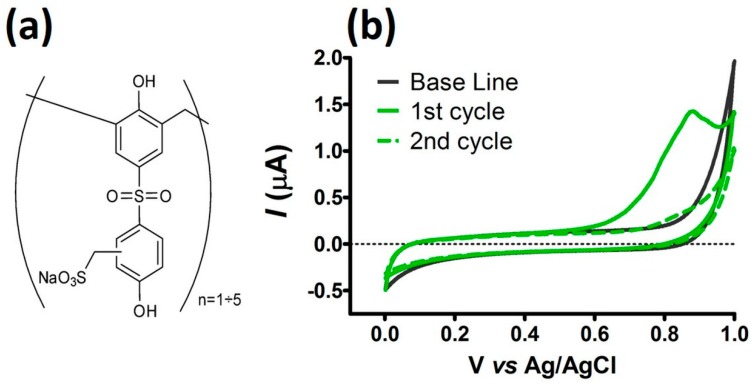
Chemical structure of Chemiplus 2DS HB (**a**). Cyclic voltammograms of Chemiplus 2DS HB on Pt-Ir in phosphate buffer (PBS, pH 7.4) (100 mV/s) (**b**): a base-line of current registered in phosphate-buffered saline (PBS) is shown as a solid dark grey line; a solid green line represents currents registered when 60 µl of ChemiPlus 2DS HB was added into the electrochemical cell (1st cycle); the dotted green line is the current registered for the subsequent cycle (2nd cycle).

Chemiplus 2DS HB, whose chemical structure is reported is part A of Figure 1, is a commercial pre-polymer, produced and supplied by BI-QEM Specialties SpA (BI-QEM Group), used as starting material to synthetize replacement syntans [22]. It is mainly employed during the tanning process in order to obtain white and pastel leather or for bleaching of wet-blue leather [23]; its use is well known on chrome-tanned leather or during vegetable retannage to get leathers with excellent fullness and firmness, and that are of a fine grain. Thanks to sulfones bridges, it allows one to have a good tanning abilities and high light fastness [24]. We postulated that Chemiplus 2DS HB bearing both a sulfone and a sulfonate group can modulate electrostatic and permeability properties of the film produced after electro-polymerization.

Research in new applications of commercially available compounds is in line with the principles of sustainable chemistry; hence, the use of Chemiplus 2DS HB in electrochemistry will aid the construction of green potentiometric and amperometric sensors. The aim of the present work is to consider the features of Chemiplus 2DS HB in sensor device construction and to evaluate the maintenance of film properties over time and under different storage conditions.

## 2. Materials and Methods

### 2.1. Chemicals

All chemicals were analytical reagent grade or higher purity and dissolved in bidistilled deionized water (MilliQ^®^). Ascorbic acid (AA), (L)-dehydroascorbate (DHA), hydrogen peroxide (H_2_O_2_), hydrochloric acid (HCl), potassium chloride (KCl), and potassium hexacyanoferrate(III) (K_3_[Fe(CN)_6_]) were purchased from Sigma-Aldrich (Milano, Italy). The phosphate-buffered saline (PBS, 50 mM) solution was prepared using 0.15 M NaCl, 0.04 M NaH_2_PO_4_, and 0.04 M NaOH from Sigma–Aldrich and then adjusted to pH 7.4.

Chemiplus 2DS HB consist of a water-soluble blend of oligomers industrially obtained by formaldehyde polymerization with 4-4ʹ dihydroxydiphenyl sulfone [25]. Bisphenol S synthesis has been well known since the 70s [26]. In the first step, phenol reacts with concentrated sulfuric acid (molar ratio phenol:sulfuric acid = 3:1) at 120 °C to obtain phenolsulfonic acid and unreacted phenol mixture. Then, distillation occurs at 150–170 °C under vacuum (−0.900 bar; distilled phenolic water is recovered and reused in other industrial processes). Distillation process allows phenolsulfonic acid to react with phenol to give 4-4′ dihydroxydiphenyl sulfone. In the second step, condensation reaction with formaldehyde and sodium bisulfite is carried out to obtain the final product. Chemiplus 2DS HB water solubility is conferred by -SO_3_H groups, resulting from sodium bisulfite linkage to the polymer structure. Chain endings are both represented by -CH_2_OH and -H [27].

Chemiplus 2DS HB molecular structure is reported in part A of Figure 1: at the most, five monomeric units are present in the oligomeric chain, with a prevalent abundance of n = 3 chains. The product is not stable in acidic media and is commercialized as sodium salt at 42% w/w water solution (pH = 8). Solution of H_2_O_2_ (100 mM) was prepared from a 30% v/v and used soon after preparation. Stock solution of AA (100 mM) was prepared in in 0.01 M HCl. DHA solution (1 mM) for storing was prepared immediately before use by dissolving the reagent into the appropriate amount of PBS. Solutions were kept at 4 °C when not in use. All in vitro calibrations were performed using fresh solutions under standard conditions of pressure and temperature. Teflon-coated platinum (90% Pt, 10% Ir; Ø = 125 μm) was purchased from Advent Research Materials (Eynsham, England).

### 2.2. Electrochemical Setup and Sensors Construction

All the working electrodes were prepared removing the Teflon^®^ insulation from the platinum wires in order to expose 1 mm of bare metal. Electrodepositions, calibrations, and cyclic voltammetries were carried out using the four-channel equipment (eDAQ QuadStat, e-Corder 410, eDAQ, Denistone East, Australia); all potentials are referred to Ag/AgCl electrode (RE); a stainless steel wire was the auxiliary electrode (AE). Cyclic voltammetries (CVs) of Chemiplus 2DS HB were performed in PBS (pH = 7.8) in order to investigate its electrochemical behavior. Potential sweeped from 0 to +1 V at a scan rate of 100 mV/s. After few cycles in PBS 60 µl of Chemiplus 2DS HB solution were added to electrochemical cell and stirred for few seconds. CVs of K_3_[Fe(CN)_6_] were performed in 0.1 mM KCl solution as background electrolyte at different scan rates (20, 50, 80, and 100 mV/s) from −0.25 V until 1 V. CVs of H_2_O_2_ and AA were performed in 50 mM PBS solution as background electrolyte at 100 mV/s from −0.25 V until 1 V.

Sensor construction was performed by soaking Pt electrodes into 20 mL PBS, in which Chemiplus 2DS HB concentration was 1.2% and a potential of +900 mV was applied for 30 minutes by means of constant potential amperometry. The area of the electrode was obtained by cyclic voltammetries in presence of K_3_[Fe(CN)_6_] performed at different scan rates on an electrode before and after modification with Chemiplus 2DS HB. The following version of Randles–Sevcik formula has been used from [28]:

(1) $$I_{P}=\left(2.69\times 10^{5}\right)n^{\frac{2}{3}}A_{eff}D_{R}^{1/2}v^{1/2}C_{0}$$

where *I_p_* refers to the anodic peak current, *n* is the number of electrons transferred, *A_eff_* is the active surface area of the electrode, *D_R_* is diffusion coefficient, *v* is the scan rate, and *C_0_* is the concentration of K_3_[Fe(CN)_6_]. In our condition: *C_0_* = 1.0 mM, *n* = 1, *D_R_* = 7.6 × 10^−6^ cm^2^/s. Then, the slopes for bare and modified Pt were calculated from *I_p_* vs. *v*^1/2^ plots. Slope for bare Pt was 1.466 μA (V s^−1^)^−1/2^ and for sensor modified with Chemiplus 2DS HB was 0.379 μA (V s^−1^)^−1/2^. Finally, the electro active areas were calculated. In our experiment, *A_eff_* of unmodified Pt was 0.198 mm^2^; after modification, *A_eff_* was calculated to be 0.051 mm^2^, revealing a fourfold decrease of *A_eff_* after modification.

### 2.3. Statistical Analysis

Oxidation currents were expressed in nanoampere (nA) and given as baseline − subtracted values ± standard error of the mean. H_2_O_2_ calibrations (from 0 until 100 mM) were performed on bare Pt electrodes, soon after polymerization with Chemiplus 2DS HB and repeated after storage. Then, linear regression from H_2_O_2_ calibrations was performed, and calculated slope (nA mM^−1^) and R^2^ values were reported in Table 1. Linear regression performed over AA calibrations fitted very badly, so in order to describe AA behavior in Table 1, Δ*I* values are reported instead of slope. AA rejection can be suitably evaluated as Δ*I* [29]. In this work, relative Δ*I* (Δ*I*_%_) was also used as reported in the following Equation (Equation (2)):

(2) $$\Delta I_{\%}=\frac{\Delta I_{sensor}}{\Delta I_{bare}}\times 100$$

where Δ*I*_%_ is the difference between currents measured pre- and post- injection of 1 mM AA; Δ*I_sensor_* corresponds to a Δ*I* measured on Pt sensor treated with Chemiplus 2DS HB; and Δ*I_bare_* is obtained from bare Pt. Since Δ*I*_%_ is a percentage, the statistical significance of Δ*I*_%_ was calculated with a Mann–Whitney test, a non-parametric test that does not assume a Gaussian distribution of variable and can only compare two independent groups. Mann–Whitney test works by ranking all the values from low to high, and comparing the mean rank in the two groups. AA behavior of sensors stored in different conditions is reported in Figures 4 and 5 as calibration plots (currents vs. AA concentration).

Limit of detection (LOD) and limit of quantification (LOQ) for hydrogen peroxide were calculated according to Equations (3) and (4), as described in the International Conference on Harmonisation (ICH) guidelines [30]:

(3) LOD=3.3*σ/slope

(4) LOQ=10*σ/slope

In Equations (3) and (4), σ is the standard deviation of the responses of sensors in blank samples and slope is calculated from H_2_O_2_ calibrations.

The linear and non-linear regression analyses were performed using the graphical software package Prism (GraphPad Software, San Diego, CA, USA). Statistical significance (p < 0.05) between slopes and currents was evaluated by calculating a two-tailed t-test, unpaired for separate group of sensors and paired for the same group at different times.

## 3. Results and Discussion

### 3.1. Voltammetric Characterization

In literature are available voltammetric studies conducted on two molecules chemically related to Chemiplus 2DS HB: the 4,4-dihydroxydiphenyl sulfone (bisphenol S, BPS) and hexasulfonate calix[6]arene (SCA). Under positive E_app_, BPS clearly passivated a carbon base electrodes [31]; otherwise, SCA cyclic voltammetry did not evidence a passivating process on Pt [32]. CV studies of Chemiplus 2DS HB are reported in part B of Figure 1. CV does not show any reduction activity in the range of potential used in this work (0 until +1 V), whereas a single oxidation peak at +866 mV vs. Ag/AgCl occurred when Chemiplus 2DS HB was added. The oxidation potential of Chemiplus 2DS HB is close to the value of SCA [32], but in subsequent cycles, Chemiplus 2DS HB peak disappeared together with a lowering of the current registered on electrode; even with a further injection of Chemiplus 2DS HB, the +866 mV peak did not reappear. Chemiplus 2DS HB behavior seems to be more similar to BPS, which rapidly passivated the electrode surface, whereas SCA did not show any current collapse over the Pt surface [32].

The electro-deposition of Chemiplus 2DS HB may be related to the oxidation of phenol hydroxyl group (–OH), and the formation of a non-conductive polymer would involve a phenoxyl radical formation, as reported for other phenols on Pt [33].

These CV results allowed one to set an applied potential of +900 mV vs. Ag/AgCl in order to perform the electro-deposition of Chemiplus 2DS HB on the Pt surface by constant potential amperometry (CPA). Because water dissociation occurs at potential of 1 V on the Pt electrode, negatively affecting the process, a higher applied potential could not be used.

### 3.2. Surface Modification after CPA Treatment with Chemiplus 2DS HB

**Figure 2:**
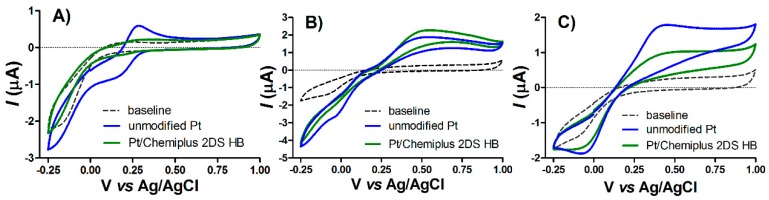
Cyclic voltammograms (range: −0.25 until 1 V) performed on unmodified Pt electrodes (blue lines) and sensor modified with Chemiplus 2DS HB (green lines) at a scan rate of 100 mV/s using different electrochemical probes: 1 mM K_3_[Fe(CN)_6_] in 0.1 M KCl as electrolyte (**A**); 1 mM of H_2_O_2_ (**B**) and 1 mM of ascorbic acid (**C**) in 50 mM phosphate buffer as electrolyte.

Further indication of surface modification are being deducted from the different behavior of microsensors against electroactive probe, as observed for the anion Fe(CN)_6_^3−/4−^ [32] and other poly-sulfone membranes [19]. Cyclic voltammetries were performed for a first qualitative assessment of surface modification; then, characterization was further improved with CPA studies. Cyclic voltammograms (Figure 2) were performed on Pt sensor before and after treatment with Chemiplus 2DS HB in presence of 1 mM of different electroactive molecules (K_3_[Fe(CN)_6_], H_2_O_2_ and AA); part A of Figure 2 shows that the anodic current of K_3_[Fe(CN)_6_] on the modified Pt decreases in respect to unmodified electrode; this further supports the actual deposition of a non-conductive Chemiplus 2DS HB film on the surface. Nonetheless, negatively charged sulfone mojeties of Chemiplus 2DS HB could be responsible for an electrostatic repulsion of the probe as found on Nafion modified glassy carbon electrode [12]. An opposite behavior can be seen for H_2_O_2_ and AA (part B and part C of Figure 2, respectively), where the oxidation of H_2_O_2_ is more intense on the modified Pt. Considering the baseline-subtracted current intensities registered at 0.7 V (the same potential used here for CPA calibration), we can appreciate a slight increment for 1 mM H_2_O_2_ oxidation (1.87 µA on Pt/Chemiplus 2DS HB vs. 1.51 µA on HP) and a 1 mM AA oxidation decrease of 50%, modified with respect to bare Pt (0.76 µA on Pt/Chemiplus vs. 1.41 µA on Pt).

Different groups of sensors obtained by CPA treatment in presence of Chemiplus 2DS HB were also probed with calibrations against H_2_O_2_ and AA and observed by scanning electron microscopy (SEM). SEM results are reported in Figure S1 of Supplementary Material, and no structural differences where evidenced between treated sensor and an unmodified Pt-Ir surface for each order of magnification. Despite this the transducer behavior with respect to H_2_O_2_, AA was sensibly altered by treatment with Chemiplus 2DS HB.

AA on unmodified Pt goes through an oxidation process prevalently by a quasi-reversible two-electron transfer process followed by chemical reaction and, to a lesser extent, through an irreversible spontaneous carbon monoxide adsorption [34]. The calibration plot of AA over bare Pt fits very well with a linear model. Analogously, we observed that under our experimental conditions a linear electro-oxidation of reporter molecule H_2_O_2_ on bare Pt was detected. The performance of Pt/Chemiplus 2DS HB was significantly altered compared with bare Pt (see Figure 2, Table 1 of main manuscript and Figures S2 and S3 of Supplementary material). Soon after the treatment with Chemiplus 2DS HB, the same group of sensors evidenced lower slopes for H_2_O_2_, while a good linear fitting (R^2^ = 0.99) of H_2_O_2_ was maintained (Table 1 and Figure S3). Although LOD and LOQ values obtained from Pt/Chemiplus 2DS HB for H_2_O_2_ are about 3 times higher when compared to poly-phenylendiamine—one of the best permselective films for H_2_O_2_ detection used in biosensor—values in Table 1 are very close to those reported for other permselective polymers [33]. A non linear behavior together with a drastic decrease in AA oxidation and Δ*I* occurred immediately after polymerization. Despite the fourfold decrease in *A_eff_* of modified sensor with respect to bare Pt calculated from Equation (1), our experiments clearly indicate that the modification of Pt surface improves H_2_O_2_ oxidation response and negatively affects AA oxidation. As seen for other polysulfone membranes, H_2_O_2_ molecules are able to penetrate the polymer matrix thanks to its small size [19]. Permeation of AA, which is larger than hydrogen peroxide, into the polymer matrix can be hindered by its dimension and by electrostatic repulsion between its deprotonated form and negative charges from -SO_3_^-^ groups from Chemiplus 2DS HB. It is acknowledged that sulfonated aromatic compounds self-assembled via the π-conjugation of benzene rings and electrostatic interactions due to hydrogen-bonding of sulfonate groups, as suggested for sulfonated-tetraphenylethylene [35]. Chemiplus 2DS HB have aromatic rings, and sulfonic acid groups are completely dissociated at pH of electro-deposition (pH 8.0), hence enabling self-assembling of polymer. It must be pointed out that the two set of sensors stored in 1 mM AA and in PBS, separately (Table 1), do not show any difference in AA or H_2_O_2_ parameters (*p* = 0.05) at day 1. Then, the set of sensors was subjected to different storage conditions, and AA and H_2_O_2_ detection were repeated after 7 days in PBS or in presence of 1 mM AA.

### 3.3. Storage Effect Characterization

#### 3.3.1. Storage Effect vs. AA Rejection: Under Constant Potential Applied

After extended storage, the H_2_O_2_ slopes of the two groups are very different (see Table 1). At day 7, H_2_O_2_ slope of sensors stored in 1 mM AA is almost 3.5 times lower than freshly prepared Pt/Chemiplus 2DS HB at day 0; meanwhile, microsensors stored in PBS have a slope similar to a bare Pt at day 0 (0.94 ± 0.21 vs. 0.76 ± 0.03). Additionally, Δ*I* parameter is significantly lower for the group stored in 1 mM AA compared to the group stored in PBS. These data suggest that polymer integrity is dramatically affected by storage conditions.

**Figure 3:**
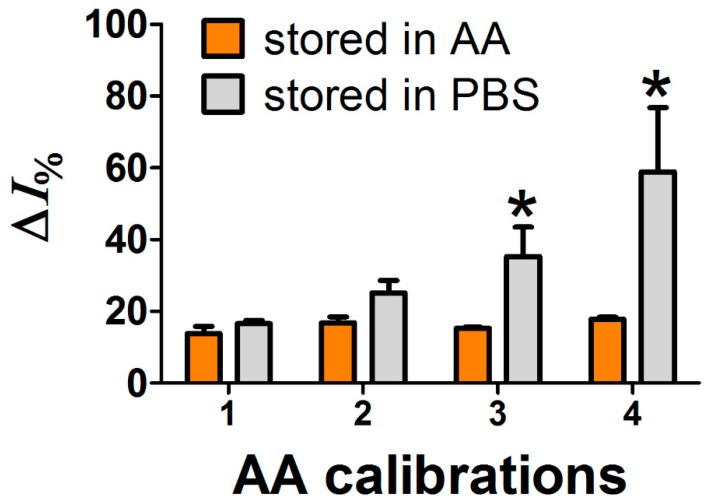
Δ*I*_%_ calculated from four calibrations of ascorbic acid (AA) are reported for two groups of sensors treated with Chemiplus 2DS HB stored in presence of AA (orange columns) or in phosphate buffer (grey columns); the AA calibrations consisted of single 1 mM AA injections into the electro-analytical cell containing a group (n = 3) of sensors; After each calibration, sensors were washed in distilled water and stored into solutions of PBS or AA with an applied potential of +700 mV until next calibration. Δ*I*_%_ is the current difference pre- and post- injection of AA reported to ΔI of bare electrode as mathematically described in statistical analysis section of material and methods. * means a statistically significant difference (*p* < 0.01).

In order to establish how much time was needed for the onset of a storage effect, two different sets of electrodes polymerized with Chemiplus 2DS HB (n = 3) were monitored for AA by time. The 1st, 2nd, and 3rd calibrations took place 1, 2, and 3 days after construction, while the 4th calibration was set after 6 days from sensors construction. During the period of calibration, sensors were kept under a constant potential of +700 mV vs. Ag/AgCl in the presence of 1 mM AA or without (PBS only); a bare Pt electrode was also included in each experimental group as internal negative control. Values of Δ*I_bare_* for experimental group stored in AA are 110 nA at 1st, 101 nA at 2nd, 126 nA at 3rd, and 109 nA at 4th. Values of Δ*I_bare_* for experimental group stored in PBS at 1st, 2nd, 3rd, and 4th calibrations are 297, 345, 267, and 256 nA, respectively. The AA rejection ability was considered as Δ*I*_%_ value (Equation (1)). For an experimental set, the electroanalytical cell solution was replaced with new PBS after each injection, and vice versa; solution was not changed in the other set, in order to leave sensors constantly immersed in 1 mM AA. In Figure 3, Δ*I*_%_ values were reported for the two sets of sensors studied. The two different storage solutions did not evidence any influence over Δ*I*_%_ until 2nd calibration; starting from 3rd calibration, a statistical significance in Δ*I*_%_ was detected, at the 4th calibration the difference in Δ*I*_%_ between the two groups almost triplicated. The AA rejection ability of sensors coated with Chemiplus 2DS HB seems to depend on their exposure to AA itself. In the group stored in PBS, the AA rejection ability seems to respond to AA injection in a different way. The longer exposure to 1 mM AA determined a stable, lower response to AA injection; meanwhile, a PBS permanence determined higher Δ*I*_%_, values, almost approaching bare Pt performance.

#### 3.3.2. Storage Effect vs. AA Rejection: Constant Potential, AA, and DHA Experiments

Since the self-induced AA rejection reveals itself between the 3rd and the 4th calibration, in the following experiments sensors were calibrated against AA at day 1 and at day 5 from construction. Sensors were stored over a range of combinations of electrochemical conditions (a constant potential applied or not) and storage solutions (Table 2). When storage was conducted under potential (E_app_), a +700 mV vs. Ag/AgCl potential was constantly applied for all the 5 consecutive days. AA is oxidized into dehydroascorbic acid (DHA) by light or oxidative potential [36]; therefore, the self-induced AA rejection could be due to both AA and its oxidative derivative DHA produced on the electrode under potential. Further experiments were carried out in order to establish if the AA rejection ability is due to a joined effect of applied potential and AA or to the sole presence of ascorbic acid.

**Figure 4:**
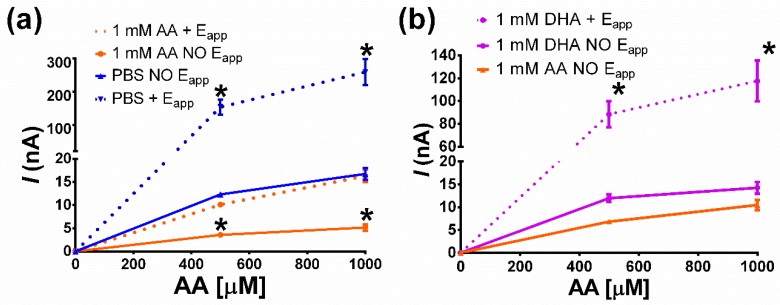
Ascorbic acid (AA) calibrations of sensors treated with Chemiplus 2DS HB (n = 4 for each experimental group) after 5 days of storage under different conditions: (**a**) treated electrodes stored in a 1 mM solution of ascorbic acid with an applied potential of +700 mV vs. Ag/AgCl (1 mM AA + E_app_), treated electrodes stored in a 1 mM solution of ascorbic acid without any potential (1 mM AA NO E_app_), and treated electrodes stored in PBS solution without any potential (PBS NO E_app_); (**b**) treated electrodes stored in a 1 mM solution of AA or dehydrodiascorbate (DHA) with an applied potential of+700 mV vs. Ag/AgCl (1 mM DHA + E_app_), treated electrodes stored in a 1 mM solution of DHA without any potential (1 mM DHA NO E_app_), and treated electrodes stored in a 1 mM solution of ascorbic acid without any potential (1 mM AA NO E_app_). A * means a statistically significant difference (*p* < 0.05) between registered currents.

**Figure 5:**
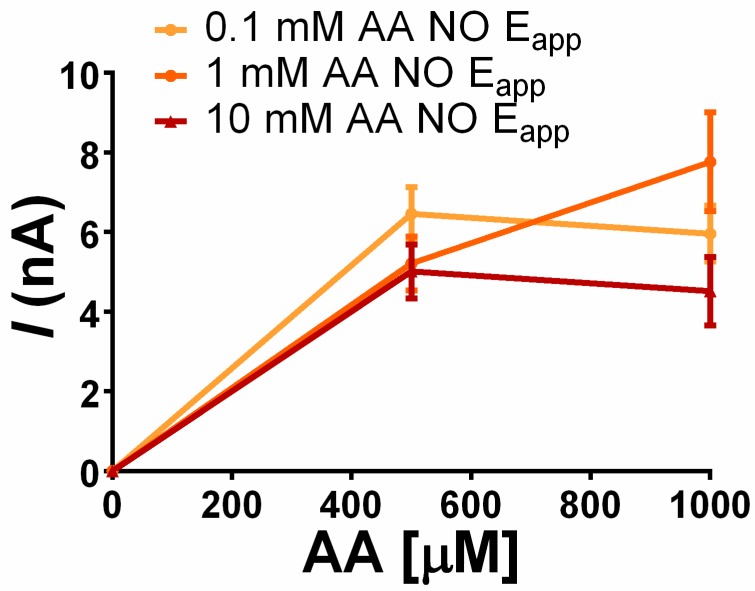
Ascorbic acid (AA) calibrations of sensors treated with Chemiplus 2DS HB (n = 4 for each experimental group) after 5 days of storage under different AA concentrations and without potential applied: treated electrodes stored in a 0.1 mM solution of ascorbic acid (0.1 mM AA NO E_app_), treated electrodes stored in a 1 mM solution of ascorbic acid (1 mM AA NO E_app_), and treated electrodes stored in 10 mM solution of ascorbic acid (10 mM AA NO E_app_).

Different sets of electrodes (n = 4) were electro-polymerized and showed homogeneous response to AA at day 1. Then, sets were stored under different experimental conditions: immersed in different solutions, applying or not a constant potential of +700 mV vs. Ag/AgCl reference electrode (AA + E_app_). The different storage conditions are reported in Table 2, and the same abbreviations are used in Figure 4 and Figure 5. At day 5, sets were washed in bi-distilled water and calibrated again in order to assess their respond to AA.

Figure 4 shows the calibration curves after 5 days under different storage conditions. Sensor sets calibrations at day 0 after calibrations, i.e., without storage, exhibited a homogeneous response to AA (data not shown). This experimental evidence confirms that differences in AA sensibility at day 5 can be preferably due to storage condition.

Part A of Figure 4 shows that the AA-rejecting phenomena in sensors that dipped in 1 mM AA occurred when potential was applied or not. Moreover, AA storage with potential and PBS with no potential were not significant (p < 0.05). This may be explained by a depletion of AA occurring when constant potential was applied during the experiment. The AA-rejecting properties were enhanced when sensors were stored in 1 mM AA at no applied potential. This could be due to a filling of polymers holes with AA molecules of the storage solution favored by the small molecular size of the acid that would hamper access to electrode or reject negatively charged AA with electrostatic repulsion. The presence of sulfone group in Chemiplus 2DS HB would improve interactions with AA molecule of the storage solution, making the filling tighter. The phenomena should be enhanced by the presence of large surface pore size and high sub-layer porosity of the electropolymerized film due to the presence of sulfonate group. DHA, the oxidation product of ascorbic acid, has no oxidative activity but is structurally similar to AA; hypothetically, DHA can occupy the same space of AA, without oxidative depletion due to applied potential. Experiments conducted with DHA are reported in part B of Figure 4: DHA with potential applied seriously affects AA rejection, as seen for PBS under potential. DHA and AA storage with no applied potential demonstrates no difference, suggesting a self-rejecting mechanism. Moreover, DHA does not have any charge, further supporting the hole-filling model.

Sulfone and sulfonate groups of Chemiplus 2DS HB would impart dimensionality to the film by creating a porous membrane, as confirmed by several authors [37,38,39,40,41]. Moreover, the hydrophilic sulfonate (R-SO_3_^-^Na^+^) group likely interacts with AA^−^ through the Na^+^ ion, probably activating interactions between the R-SO_3_- and the AA^−^ groups. As a result, the system should be more stable in a medium containing a higher concentration of AA^−^ (that is, positioned in the pores of the film created by sulfone/sulfonate groups) rather than in a solution of PBS.

Differing behavior of AA in sensors was reported in literature: AA can be absorbed in a reversible way on polarized Pt surface in H_2_SO_4_ and phosphate buffer 7.2; vice versa DHA gives no faradaic current or adsorption [36]. Suggested mechanism of oxidation of AA to DHA on Pt in acidic media was described [42].

#### 3.3.3. Storage Effect vs. AA Rejection: AA Concentration

In order to ascertain whether the AA rejection property depends on AA concentration during storage, further experiments were carried out. Figure 5 shows that there is no significant difference between AA rejection ability among sensors stored in 0.1, 1, and 10 mM AA. These experiments confirm that the peculiar AA self-rejection of the polymer can be obtained over a wide range of AA storage concentrations. As a result, the polymer can exert an effective AA rejection when sensors are maintained in matrices containing both low and high AA levels.

## 4. Conclusions

A substantial modification of Pt towards electroactive probes K_3_[Fe(CN)_6_], H_2_O_2_, and AA was successfully achieved by using the commercial oligomer Chemiplus 2DS HB. CV and CPA studies carried out before and after modification confirm that this sulfone/sulfonated-containing pre-polymer can be electrodeposited by CPA on Pt wire. Although the electroactive area of Pt/Chemiplus 2DS HB was four times smaller than bare Pt, the modified sensors retained H_2_O_2_ slopes closer to pre-modification values. On the contrary, AA rejection observed soon after polymerization was very different from bare Pt, showing a drastic decrease in Δ*I*. This peculiar feature towards AA might be due to both the aromatic structure and the presence of sulfone/sulfonate groups of oligomers able to embed AA molecules in the net. Moreover, AA and H_2_O_2_ behavior is influenced by time and storage conditions. The best AA rejection was achieved when the Pt/Chemiplus 2DS HB sensors were stored with AA solution for 7 days and no potential was required.

The sensor can provide an unexpected and straightforward way to detect AA where the presence of the analyte is mandatory. After a few days of storage without potential of Pt/Chemiplus 2DS HB sensors into the matrix, AA behavior can be evaluated in terms of Δ*I* that can be detected over a wide range of AA concentration. The maintenance of a low Δ*I* could be a signal for AA persistence into the sample, giving an advantage over the existing methodologies that require complex and expensive electrodes and applied potential during the storage.

## Figures and Tables

**Table 1:** Descriptive parameters of the behavior of groups of sensor against reporter molecule H_2_O_2_ and ascorbic acid (AA). R-squared (R^2^) evaluates the goodness of fit of the linear regression of current registered during calibration, when a bad fitting of linear model occurred R^2^ is not reported. LOD and LOQ mean limit of detection and limit of quantification, respectively. Different groups of sensor (n = 4) were assayed for H_2_O_2_ and AA before and immediately after the treatment with Chemiplus 2DS HB, then stored in presence (stored in 1 mM AA) or not (stored in PBS) of AA for 7 days under potential (+700 mV vs. Ag/AgCl) and subsequently recalibrated. Calibration plot for H_2_O_2_ behavior is reported in Figure S3 of Supplementary Material.

**H_2_O_2_ Behavior**										
**Storage Condition Group:**	**Bare Pt (Day 0)**		**After Construction (Day 0)**				**After Storage (Day 7)**			
	**Slope (nA µM^−1^)**	**R^2^**	**Slope (nA µM^−1^)**	**R^2^**	**LOD (µM l^−1^)**	**LOQ (µM l^−1^)**	**Slope (nA µM^−1^)**	**R^2^**	**LOD (µM l^−1^)**	**LOQ (µM l^−1^)**
Stored in 1 mM AA	0.83 ± 0.03	0.99	0.52 ± 0.01	0.99	0.19 ± 0.03	0.65 ± 0.07	0.15 ± 0.02	0.99	0.55 ± 0.06	1.85 ± 0.31
Stored in PBS	0.76 ± 0.03	0.99	0.51 ± 0.02	0.99	0.17 ± 0.03	0.56 ± 0.06	0.94 ± 0.21	0.98	0.06 ± 0.01	0.20 ± 0.04
**AA Behaviour**										
**Storage Condition Group:**	**Bare Pt (Day 0)**		**After Construction (Day 0)**				**After Storage (Day 7)**			
	**Δ*I* (nA)**	**R^2^**	**Δ*I* (nA)**		**R^2^**		**Δ*I* (nA)**		**R^2^**	
Stored in 1 mM AA	185 ± 5	0.99	6.10 ± 2.93		-		8.55 ± 4.46		-	
Stored in PBS	182 ± 10	0.99	2.25 ± 0.53		-		99.3 ± 23.7		-	

**Table 2:** Different storage conditions used for evaluating sensor behavior; sensors set names correspond to Figure 3 and Figure 4 legends.

Sensors Set Name (n = 4)	Storage Solution	Constant Potential Applied (+700 mV vs. Ag/AgCl)
PBS + E_app_	Phosphate buffer (PBS)	YES
PBS NO E_app_	Phosphate buffer (PBS)	NO
1 mM AA + E_app_	PBS + 1 mM Ascorbic Acid	YES
10 mM AA NO E_app_	PBS + 10 mM Ascorbic Acid	NO
1 mM AA NO E_app_	PBS + 1 mM Ascorbic Acid	NO
0.1 mM AA NO E_app_	PBS + 0.1 mM Ascorbic Acid	NO
1 mM DHA + E_app_	PBS + 1 mM Dehydroascorbic Acid	YES
1 mM DHA NO E_app_	PBS + 1 mM Dehydroascorbic Acid	NO

## Supplementary Materials

The following are available online at https://www.mdpi.com/1424-8220/19/11/2435/s1, Figure S1: micrographs obtained with a conventional scanning electron microscope of sensor surface functionalized with ChemiPlus 2 DS HB at 2000x (C1), 5000x (C2) and 10000x (C3) of magnification compared with a bare Platinum-Iridium surface at 2000x (Pt1), 5000x (Pt2), and 10000x (Pt3). Figure S2: cyclic voltammograms (range: −0.25 until 1 V, scan rate of 100 mV s^−1^ ) performed in 50 mM phosphate buffer as electrolyte on unmodified Pt electrodes (blue lines, part A and C) and sensor modified with Chemiplus 2DS HB (green lines, part B and D) using different electrochemical probes 1 mM of H_2_O_2_ (part A and B) and 1 mM of ascorbic acid (part C and D). Figure S3: constant potential amperometry calibration plots for of H_2_O_2_ performed at day 0 on sensors modified with Chemiplus 2DS HB (black lines) and performed ad day 7 after storage in 1 mM ascorbic acid (AA) (orange line in part A) and after 7 days of storage in PBS (blue line in part B). These graphics correspond to values reported for H_2_O_2_ behavior of Table 1 in the main manuscript. (n = 4 for each experimental group, solid line comes from linear regression of currents, and vertical bars are standard error of the mean; each storage group was subjected at a +0.7 V of potential applied).
