# Supplementary material for: A Storage-Dependent Platinum Functionalization with a Commercial Pre-Polymer Useful for Hydrogen Peroxide and Ascorbic Acid Detection
Source: Sensors (Basel). 2019 May 28;19(11):2435. doi: 10.3390/s19112435 (PMC6603770; doi:10.3390/s19112435)
Supplement: Supplementary file 1 [file sensors-19-02435-s001.pdf]

Article

A Storage-Dependent Platinum Functionalization with a Commercial Pre-Polymer Useful for Hydrogen Peroxide and Ascorbic Acid Detection

Patrizia Monti [1], Quirico Migheli [1], Andrea R. Bartiromo [2], Antonio Pauciulo [2], Rocco Gliubizzi [2,*], Salvatore Marceddu [3], Pier A. Serra [4,*] and Giovanna Delogu [5]

1. Dipartimento di Agraria and Unità di Ricerca Istituto Nazionale di Biostrutture e Biosistemi, Università degli Studi di Sassari, Viale Italia 39, I-07100 Sassari, Italy; pmonti@uniss.it (P.M.); qmigheli@uniss.it (Q.M.)
2. R&D Department – Zona Industriale, BI-QEM Specialties SpA, I-84021 Buccino (SA), Italy; andrea.bartiromo@bi-qem. (A.R.B.); comantonio.pauciulo@bi-qem.com (A.P.)
3. Istituto CNR di Scienze delle Produzioni Alimentari, sede di Sassari, Traversa La Crucca 3, I-07100 Sassari, Italy; salvatore.marceddu@ispa.cnr.it
4. Dipartimento di Medicina Clinica e Sperimentale, Università degli Studi di Sassari, Viale S. Pietro 43/b, I-07100 Sassari, Italy; paserra@uniss.it
5. Istituto CNR di Chimica Biomolecolare, sede di Sassari, Traversa La Crucca 3, I-07100, Sassari, Italy; giovanna.delogu@icb.cnr.it

* Correspondence: rocco.gliubizzi@bi-qem.com (R.G.); paserra@uniss.it (P.A.S.); Tel.: +39-0828-957272 (R.G.); Tel.: +39-079-228-558 (P.A.S.)

Received: 15 April 2019; Accepted: 24 May 2019; Published: date

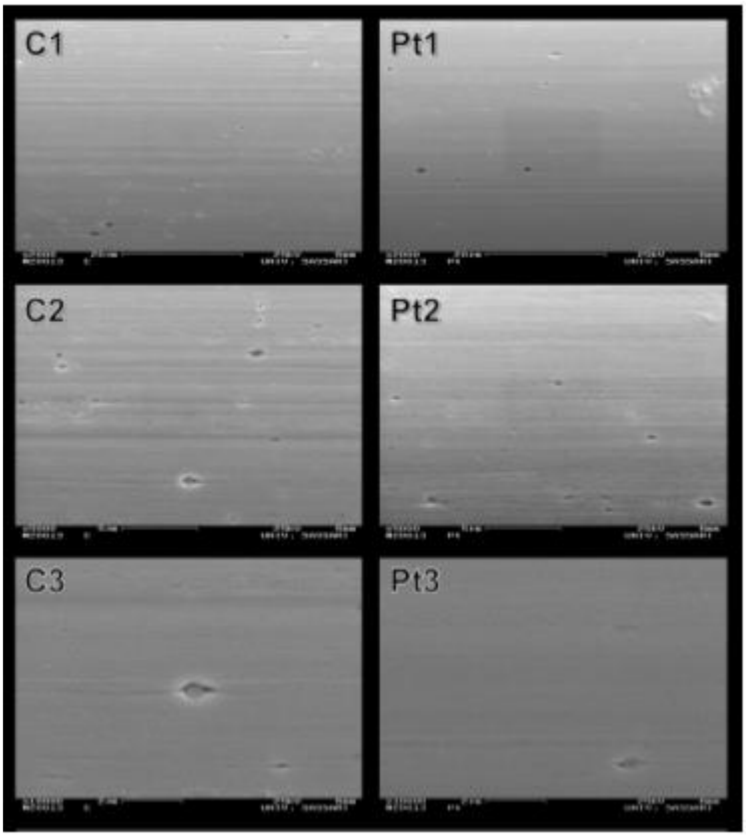

Figure 1. micrographs obtained with a conventional scanning electron microscope of sensor surface functionalized with ChemiPlus 2 DS HB at 2000x (C1), 5000x (C2) and 10000x (C3) of magnification compared with a bare Platinum-Iridium surface at 2000x (Pt1), 5000x (Pt2), and 10000x (Pt3).

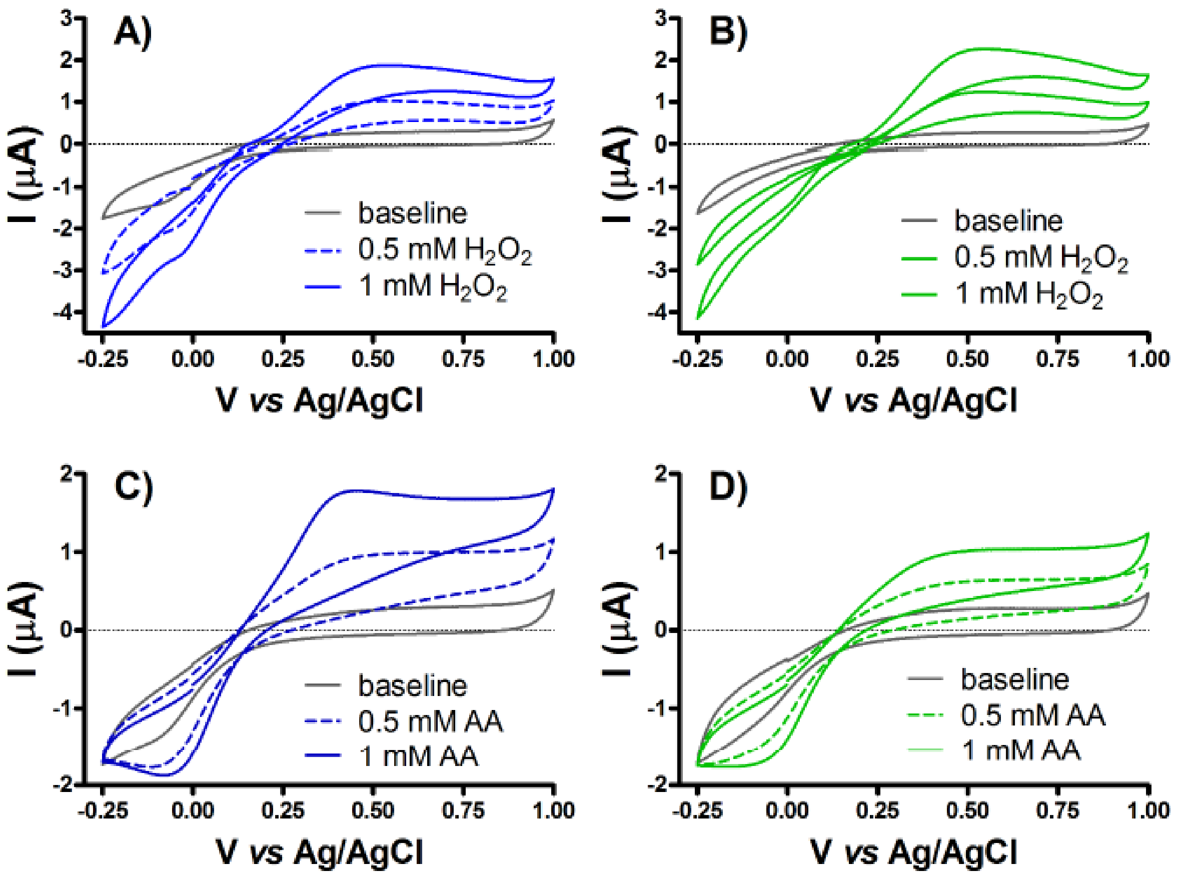

Figure 2. cyclic voltammograms (range: −0.25 until 1 V, scan rate of 100 mV s^{-1}) performed in 50 mM phosphate buffer as electrolyte on unmodified Pt electrodes (blue lines, part A and C) and sensor modified with Chemiplus 2DS HB (green lines, part B and D) using different electrochemical probes 1 mM of H_2O_2 (part A and B) and 1 mM of ascorbic acid (part C and D).

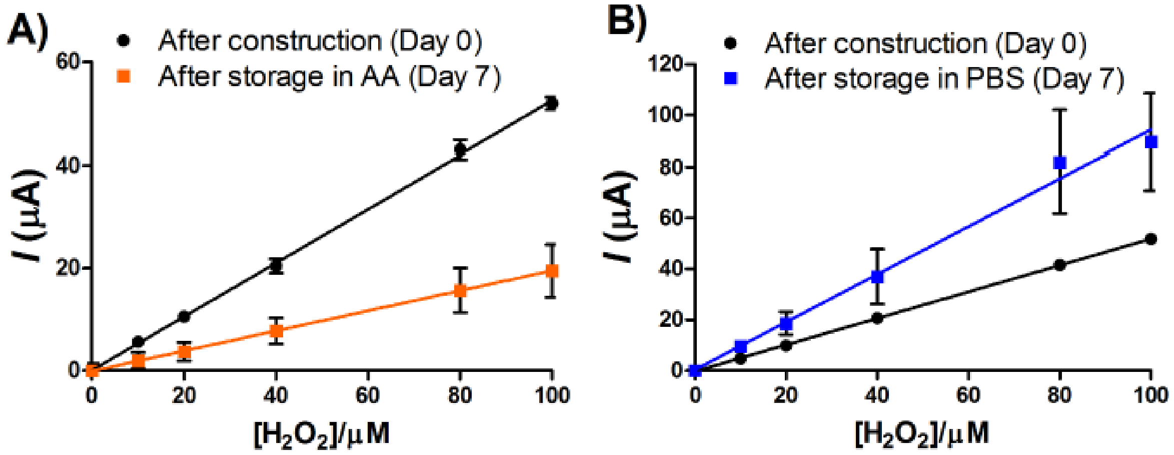

Figure 3. constant potential amperometry calibration plots for of H_2O_2 performed at day 0 on sensors modified with Chemiplus 2DS HB (black lines) and performed ad day 7 after storage in 1 mM ascorbic acid (AA) (orange line in part A) and after 7 days of storage in PBS (blue line in part B). These graphics correspond to values reported for H_2O_2 behavior of Table 1 in the main manuscript. (n = 4 for each experimental group, solid line comes from linear regression of currents, and vertical bars are standard error of the mean; each storage group was subjected at a +0.7 V of potential applied).
